# Prognostic value of the immunohistochemical expression of vascular endothelial growth factors in malignant salivary gland neoplasms: a systematic review and meta-analysis

**DOI:** 10.4317/medoral.23974

**Published:** 2021-02-20

**Authors:** Erison Santana dos Santos, Joab Cabral Ramos, Ana Gabriela Costa Normando, Adriana Franco Paes Leme

**Affiliations:** 1 DDS, MSc, PhD Student. Department of Oral Diagnosis, Piracicaba Dental School, University of Campinas, Piracicaba, Brazil; 2 DDS, MSc, PhD, Professor. Brazilian Bioscience National Laboratory, Brazil Center of Research in Energy and Materials, Campinas, Brazil

## Abstract

**Background:**

The immunohistochemical expression of vascular endothelial growth factor is a prognostic marker in several cancer types. In salivary gland tumors, the association between vascular endothelial growth factor and prognosis remains unclear. The purpose of this study was to perform a systematic review and meta-analysis to assess whether the immunohistochemical expression of vascular endothelial growth factor in patients with salivary gland neoplasms presents prognostic value.

**Material and Methods:**

Immunohistochemical studies assessing the predictive value of vascular endothelial growth factor in salivary gland neoplasms were systematically reviewed using PubMed, Scopus, Embase, Cochrane Library, and Web of Science databases. It was assessed any survival rates. The fixed-effect model with an adjusted hazard ratio (HR) and 95% confidence intervals (95% CI) as effect measures were performed in the meta-analysis. The Quality in Prognosis Studies (QUIPS) tool was used to assess the quality of the included studies, and the evidence quality was assessed by the Grading of Recommendation, Assessment, Development, and Evaluation (GRADE) system.

**Results:**

The immunohistochemical overexpression of vascular endothelial growth factor in patients with salivary gland neoplasms was associated with shortened survival (HR=5.37, 95% CI: 2.67-10.83, *P* = 0.00001). In addition, the presence of vascular endothelial growth factor was tightly associated with tumor size, lymph node metastasis, clinical stage, perineural invasion, vascular invasion, poor local control of the disease, and recurrence.

**Conclusions:**

The immunohistochemical overexpression of vascular endothelial growth factor in patients with salivary gland neoplasms has prognostic value and was associated with decreased survival time. However, more primary well-designed studies are necessary to increase the level of evidence.

** Key words:**Salivary gland neoplasms, salivary glands, head and neck neoplasms, vascular endothelial growth factors, prognosis.

## Introduction

Angiogenesis is defined as the development of new blood vessels from the preexisting vascular beds and plays an essential role in the tumor's maintenance, growth, and progression (1). A range of molecules such as cytokines, proteins, and growth factors tightly regulate this process (1,2). Among various angiogenic factors, the most studied is the vascular endothelial growth factor (VEGF) due to its capacity to initiate and regulate the process of angiogenesis. This biologic event is complex, and it has been shown that hypoxia plays an essential role in the initiation of angiogenesis (1-3). The lack of oxygen leads to the tumor's cells release pro-angiogenic factors such as VEGF (3). Besides, it has been demonstrated that hormones, growth factors, hypoglycemia, and altered expression of genes are capable of upregulating VEGF expression (3).

In the context of head and neck cancer, it has been demonstrated that VEGF overexpression is associated with poor overall survival (4). Nevertheless, it is still uncertain whether VEGF overexpression is a risk factor for shorter survival in patients with salivary gland neoplasms (SNGs). Salivary gland neoplasms are a diverse group of tumors with different clinical, biological, and molecular features that correspond to approximately 3% to 10% of the tumors of the head and neck region (5). Several studies have attempted to determine the prognostic importance of VEGF in these lesions; however, the results obtained are conflicting (1,4,5). Therefore, the aim of this systematic review and meta-analysis was to assess the prognostic value of VEGF in patients with malignant SGNs.

## Material and Methods

- Protocol and registration:

A search was carried out for any registered protocols on a similar topic in the International Prospective Register of Systematic Reviews (PROSPERO). No systematic review protocols were found in this database. Therefore, this review was registered in the PROSPERO platform under the identification number CRD42020181985. The systematic review was reported according to the Preferred Reporting Items for Systematic Reviews and Meta-Analyses (PRISMA) statement (6).

- Eligibility criteria

It was established a PECOS framework (Population, Exposure, Comparison, Outcomes, and Studies) according to PRISMA, that was used to formulate the focused question of the review, of which: P) patients with the diagnosis of malignant salivary gland neoplasms; E) analysis by immunohistochemical detection of VEGF C) patients with no history of malignant salivary gland neoplasms; O) survival analysis; S) observational studies (case-control, cross-sectional, or population-based) and/or randomized clinical trials. Articles were excluded by the following reasons: A) did not fit PECOS strategy; B) did not present prognostic value of VEGF on SGNs such as hazard ratio (HR) values and corresponding 95% confidence intervals (CIs), disease-free survival (DFS), disease-specific survival (DSS), overall survival (OS) or any other survival analysis; C) *in vitro* or *in vivo* experimental studies, letters to the editor, short communications, personal opinions, conference abstracts, case reports, and reviews.

- Focused question

Is there any association between immunohistochemical detection of VEGF and overall survival in patients with malignant salivary gland neoplasms?

- Search strategy

A search was conducted on April 24, 2019, and adapted for each electronic database: PubMed, Scopus, Web of Science, Embase, and Cochrane Library. An additional gray literature search was performed on Google Scholar, Open Grey, and ProQuest Dissertation & Theses Global. The search strategy was based on combinations of the following keywords: ("Vascular Endothelial Growth Factors"[MeSH] OR "VEGFs") AND ("Salivary Gland Neoplasms"[MeSH] OR "Salivary Gland Neoplasm" OR "Cancer of the Salivary Gland" OR "Salivary Gland Cancers" OR "Cancer of the Salivary Gland" OR "Salivary Gland Cancer" OR "Salivary Glands"[MeSH] OR "Salivary Gland"). All duplicate references were removed using a reference manager software (Rayyan QCRIR) (7). In addition, manual screening of the reference lists from the selected articles was performed to identify potentially relevant studies that could have been missed during the electronic database searches.

- Study selection and data extraction

The process of study selection was performed in two phases. First, titles and abstracts of all identified articles were screened by two independent reviewers (ESS and AGCN) using a standardized guide. This stage was conducted using Rayyan—a web and mobile app for systematic reviews available online (7). The same two authors read the full texts of the selected articles at phase one and excluded those that did not meet the inclusion criteria. Cohen's Kappa analysis was performed to a quantified agreement between the authors. Another author (AFPL) was involved in the case of doubts and conflicts. Two reviewers (ESS and AGCN) independently collected the data on study characteristics such as author, year of publication, country of the first author of the study, the number of samples enrolled, histologic type of SGN, follow up period, statistical method, and main results.

- Quality assessment

The recommendations of the Cochrane Prognosis Methods Group were followed and the Quality in Prognosis Studies (QUIPS) tool was used to assess the methodological quality of included studies (8,9). The QUIPS tool assesses the risk of bias in prognostic studies by rating each article in six domains: study participation, study attrition, measurement of prognostic factors, measurement of outcomes, measurement of confounding, and statistical analysis and reporting. A judgment of the risk of bias on each domain of the tool was made from the extracted information, rated as “high,” “moderate,” or “low” risk. These judgments were realized by two authors (ESS and AGCN), independently and blindly. In case of disagreements, a third author (AFPL) was consulted.

- Summary of measures and synthesis of results

It was performed a meta-analysis of the survival rates following the appropriate Cochrane Guidelines for prognostic reviews (10). Review Manager 5.3 (RevMan 5.3, The Nordic Cochrane Centre, Copenhagen, Denmark) was used to construct the forest plots of the meta-analysis. The HR and 95% CI were used to determine at a significance level of 5%, according to the adjusted survival rates original values of the included articles. The heterogeneity between eligible studies was calculated by inconsistency indexes (I2). I2 > 50% were considered indicators of substantial heterogeneity. In the case of no significant heterogeneity, a fixed-effects model was used. A P-value < 0.05 was considered statistically significant.

- Risk of bias across studies

Clinical heterogeneity was assessed by comparing variability among the number of samples and outcomes for survival studies; methodological heterogeneity was assessed by the risk of bias and variability in the study design. Statistical heterogeneity was also considered (HR and 95% CI).

- Confidence in cumulative evidence

The Grading of Recommendation, Assessment, Development, and Evaluation (GRADE) instrument was used to assess evidence quality and grading of recommendation strength in the five studies included in the quantitative synthesis (11). This assessment was based on the study design, risk of bias, inconsistency, indirectness, imprecision, and other considerations. Evidence quality was characterized as high, moderate, low, or very low. The GRADE was assessed using the website http://gradepro.org.

## Results

- Study Selection

**Figure 1:**
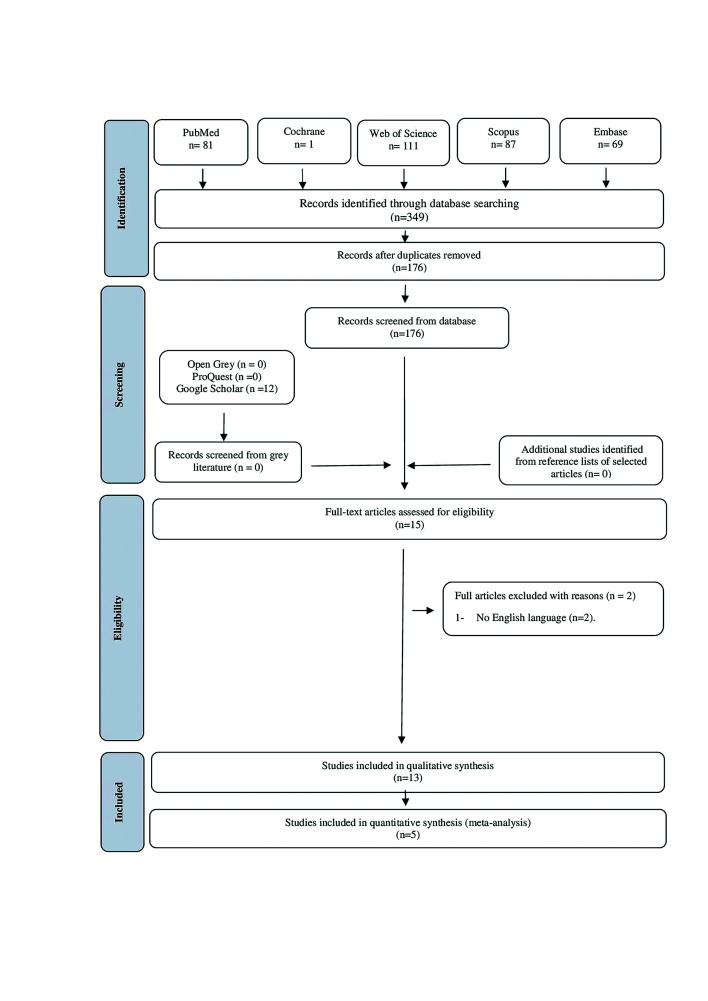
Flow diagram of literature search and selection criteria.

In the first stage of this review, 349 studies were found in the five databases. After duplicate articles were removed, 176 remained. A screening of the titles and abstracts was carried out, and 15 records moved on to the second selection phase. In the second stage, the full-text review was then conducted on the 15 first stage selected studies, which led to the exclusion of 2 studies. After this, 13 studies fulfilled the inclusion criteria and were included in the systematic review. However, only 5 of these studies performed multivariate analysis, and the adjusted HR and 95% CI was accessible, enabling quantitative analysis. Cohen's Kappa analysis overall score was 0.81. Fig. 1 details this process of study selection.

- Studies Characteristics

Among the included studies, four were from China (1,12-14), three were from South Korea (15-17), two from Spain (18,19), and one each from Austria (20), Brazil (5), Czech Republic (21), and Japan (22). All included studies were observational studies. The year of publication of included articles ranged from 1999 to 2016. The total number of samples was 861 (775 SGNs and 86 controls). Adenoid Cystic Carcinoma (ACC) was the most studied neoplasm, followed by Mucoepidermoid Carcinoma (MEC). The follow-up period ranged from 1 to 600 months. According to the statistical analysis, the Kaplan-Meier method was used to calculate survival curves in all included studies. Univariate and multivariate Cox proportional hazard regression analysis was applied in six studies (1,12,15-17,19). However, six studies did not perform Cox regression due to the absence of significance in the log-rank test and univariate analysis (5,13,14,18,20,21), and in one study, the test applied was not mentioned (22). The main features and findings of the studies are presented in Table 1.

- Risk of bias within studies

**Figure 2:**
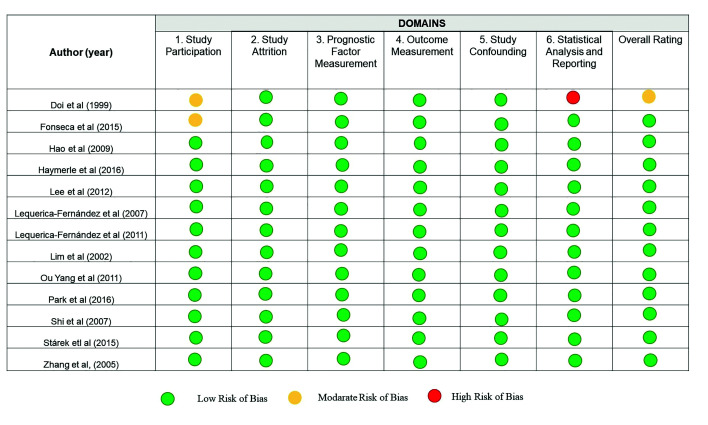
Risk of bias of included studies according to the Quality In Prognosis Study (QUIPS) tool.

Twelve studies were classified as an overall low risk of bias, and one was graded as overall moderate risk. Among the thirteen studies included, eleven studies obtained a low-risk rate on all domains (1,12-21). Two articles were graded as moderate risk on the study participation domain (5,22). Of these two studies, one was graded as a high risk for statistical analysis and reporting (22). Among these two articles, there was a lack of adequate description of inclusion and exclusion criteria (5,22), and the test used for survival analysis was not mentioned (22). Details about this process are shown in Table 2. The overall risk of bias assessment of the thirteen included studies is summarized in Fig. 2.

- Results of individual studies

- Demographic features

In two studies, males predominated, while nine studies demonstrated a female predilection. The F:M ratio among the included studies was 1.19:1, of which females were more affected [399] than males [335]. Regarding the age, it was observed a range from 13 to 91 years old. The most affected site was major salivary glands (456 cases) followed by the minor salivary gland (319 cases). The most affected subsite was the parotid gland (285 cases). Glands from other sites such as the paranasal sinuses, nasopharynx, nasal cavity, larynx, and the auditory canal were included in some studies.

**Table 1:**
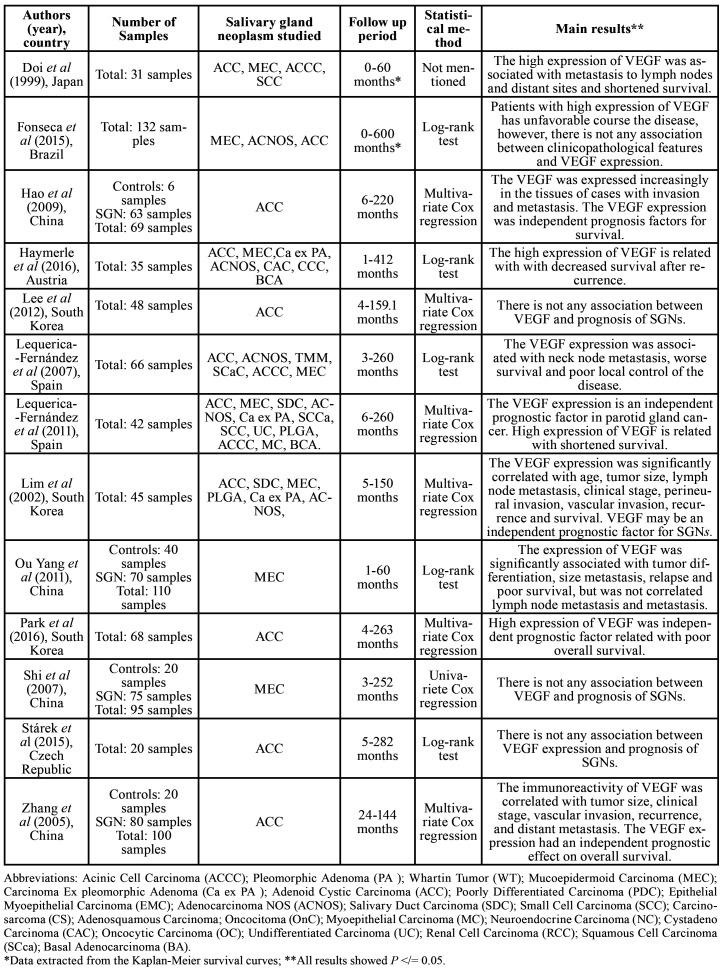
Key features and main findings of studies concerning the prognostic value immunohistochemistry detection of VEGF in SGNs.

**Table 2:**
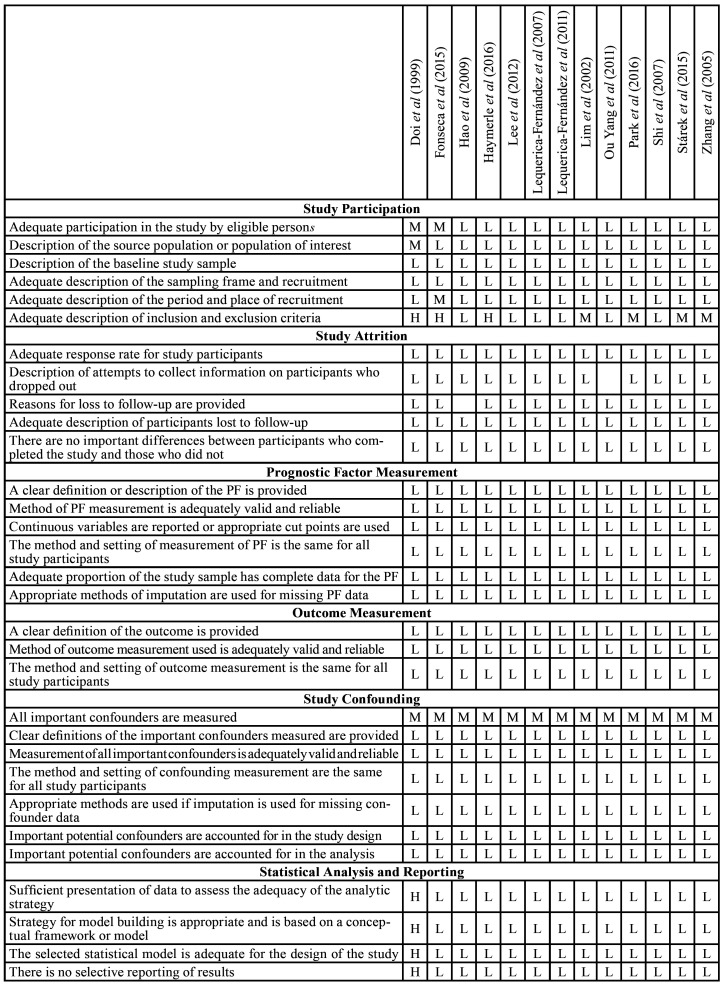
Risk of bias categorized as (H) High risk, (L) Low Risk and (M) Moderate Risk according to recommendations from Cochrane Methods Prognosis group utilizing the Quality in Prognosis Studies (QUIPS) tool.

- Microscopic features

All included studies provided a histologic classification of their samples, and they graded the tumors according to the classification of the World Health Organization (WHO) (23). Adenoid cystic carcinoma was by far the most studied lesion followed by mucoepidermoid carcinoma. The histologic patterns of ACC included were cribriform, tubular, solid, and intermediate. MEC were classified as low-grade, intermediate-grade, and high-grade. Eight studies reported the presence of perineural invasion in a sample of 153 patients. Vascular invasion was reported in four studies affecting 50 patients.

- Clinical features

The presence of clinical symptoms, such as numbness, pain, bleeding, and paresthesia, was observed in 96 patients. Regarding the clinical stages, 300 patients were classified on clinical stage I/II and 245 patients in clinical stage III/V. -

- Overall analysis of VEGF expression

VEGF was expressed both in the cytoplasm and in cell membranes with different intensities. Immunoreactivity was present in 87% [676] of cases analyzed. Despite heterogeneity among the histopathologic types of SGNs analyzed, some studies demonstrated that the VEGF was associated with shortened overall survival in patients with SGNs. On the other hand, some studies showed a lack of association between VEGF expression and any prognostic factor in patients with SGNs.

The tumor that showed the greatest association with increased VEGF expression was ACC. In the studies that analyzed ACC isolated, it was shown that overexpression of VEGF was significantly associated with decreased survival and was correlated with tumor size, clinical stage, vascular invasion, recurrence, and distant metastasis (P < 0.05) (4,12,17). VEGF was not associated with age, tumor sites, and patients ‘gender. Despite this, two studies failed to find an association between VEGF overexpression and survival (14,15). Some studies correlated the VEGF expression and MEC, and it was observed that the expression levels of VEGF were significantly related to tumor differentiation, size, and relapse (P < 0.05), but were not correlated with lymph node metastasis, distant metastasis, or overall survival (13,14).

Among the studies that pooled different types of SGNs, there was a strong association of clinical and pathologic factors and VEGF expression, such as clinical stage, tumor size, age, vascular invasion, perineural invasion, survival, and recurrence (P < 0.05) (16,18-20). The isolated prognostic factor strongly associated with different levels of VEGF expression was metastasis. In general, there was a significant association between immunohistochemistry overexpression of VEGF and shortened survival.

- Synthesis of results

**Figure 3:**
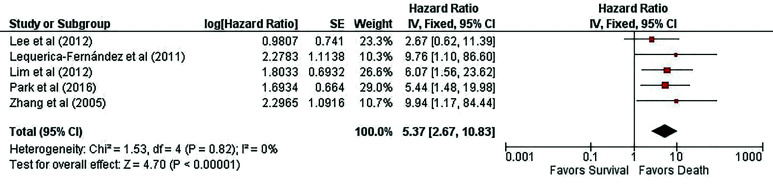
High expression of VEGF is significantly associated with shorten survival. Forest plot of hazard ratio for survival comparing patients with overexpression of VEGF in the malignant salivary gland tumors compared with those with low immunoreactivity. The meta-analysis revealed that VEGF was associated with poorly survival (HR: 5.37, 95% CI: 2.67-10.83, P < .00001). The diamond represents the pooled HR performed by the fixed-effect model.

Five studies assessed the OS of SGN samples using Kaplan-Meier curves, which generated Hazard Ratios (HR). The meta-analysis used the adjusted hazard ratio + 95% CI was derived from the multivariate analysis from included articles. The pooled HR was 5.37 (95% CI: 2.67-10.83; P = 0.00001; I2 = 0%). Low heterogeneity was observed between the studies, with an I2 of 0% (*P*=0.82), leading to the decision of the fixed-effect model. As shown in Fig. 3, higher immunohistochemical expression of VEGF in SGNs may favor shortened overall survival. One study found a significant association between VEGF's high immunohistochemical expression and poor overall survival, and it was an independent prognostic factor (17).

- Risk of bias across studies

Regarding the risk of bias across studies, the selected studies used similar methods, which reduced the possibility of misinterpretation. According to studies design, they were considered homogeneous, which was confirmed by the low heterogeneity that led to the fixed effect choice.

- Confidence in cumulative evidence

According to GRADE analysis, the quality of the evidence for survival analysis was moderate. Due to this, it is reasonable to suggest moderate confidence in estimating the outcomes. The CI difference in each study was the main factor responsible for the limited quality of evidence on the imprecision assessment (Table 3).

**Table 3:**
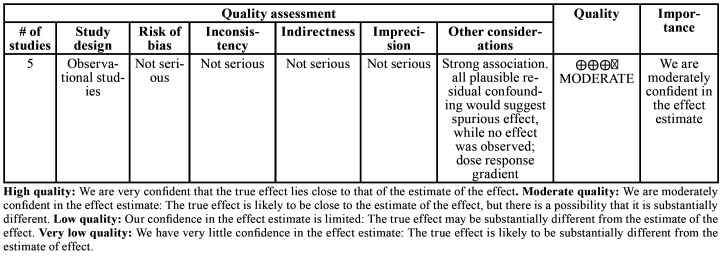
Table representing prognostic studies included in the meta-analysis assessing Grading of Recommendation, Assessment, Development, and Evaluation (GRADE) instrument. Question: Is there any association between immunohistochemical detection of VEGF and overall survival in patients with malignant salivary gland neoplasms?

## Discussion

The role of VEGF in tumor progression and metastasis has been studied in several malignant neoplasms, including head and neck cancer (1,4,5). Despite this, the data of the prognostic value of VEGF in patients with SGNs remains unclear. Some studies have reported that VEGF may be useful as an independent prognostic factor for these tumors (1,15-17,19). On the other hand, some authors suggested that there is not any significant association between VEGF and SNGs (5,14,15,21). Due to these controversial findings, we performed a systematic review and meta-analysis from the literature to assess the association of VEGF by immunohistochemical analysis and the overall survival of patients with SGNs. Data were extracted from thirteen studies with 775 samples of SGNs tissue analyzed by immunohistochemistry. The combined results showed that the positivity for VEGF in the tissue of these patients was associated with shortened survival. Moreover, the presence of VEGF was associated with clinical stage, perineural invasion, vascular invasion, tumor size, poor local control of the disease, and recurrence (1,13,16,18). In addition, publication bias examination and heterogeneity assumption tested by the I2 metric support our findings, although, due to CI difference in each study, the quality of the evidence for survival analysis was moderate.

The prognostic factor more associated with VEGF overexpression was metastasis (1,12,13,16,18,22). Metastasis is an event uncommon on salivary gland tumors, and its mechanism is still unclear in these lesions. However, it is well established that patients with local and distant metastasis have a poor prognosis (4). It is not fully understood how VEGF can promote metastasis in SNGs, but this relation was largely studied in other tumors (24). The activation of VEGF family members and their receptors aid in the escape of the immune system, migration, and extracellular matrix invasion promoting metastasis (24). In this systematic review, it was observed that patients with high levels of VEGF expression presented distant or lymph node metastasis and poor prognosis.

Interestingly, four studies did not find any association between VEGF and prognosis in SGNs (5,14,15,21). Fonseca and colleagues pooled a total of 132 formalin-fixed, paraffin-embedded tissue of SGNs (5). They failed to identify an association between clinicopathologic features and VEGF expression, probably due to the small sample size of malignant SGNs analyzed in the study. This finding was confirmed by other authors, although the majority of samples analyzed were composed of tumors of clinical stages I and II and lower histologic grade (14). SGNs are a heterogeneous group of lesions with different molecular mechanisms involved in its progression. The angiogenesis may be affected by each tumor's inherent features, leading to different expression of VEGF.

The prognostic value of VEGF has already been evaluated in other types of cancer, such as colorectal and non-small cell lung cancer (NSCLC) (25,26). In a previous study, 1.428 colorectal cancer patients from 13 clinical trials were pooled in a meta-analysis, and it was observed that immunohistochemical detection of VEGF predicted poor survival in these patients (25). Also, VEGF's immunohistochemical expression was associated with poor prognosis for NSCLC patients, including patients with clinical stage I (26). However, VEGF was not significantly correlated with survival for patients with lung adenocarcinoma (26). Interestingly, our results showed a close association of VEGF overexpression in adenocarcinoma of salivary glands and poor survival (16,18-20). To the best of our knowledge, this current systematic review and meta-analysis was the first to find an association between VEGF and prognosis in SGNs. These findings confirm the results of previous studies and lead to the necessity of more investigation of the role of VEGF in SGNs.

It is necessary to highlight some limitations in this review. First, although only one technique was used in all studies (immunohistochemistry), the variation in antibodies utilized in each study may lead to differences in the quality and intensity of staining results and interpretation of results. Second, the studies analyzed a limited sample size of SGNs, and some studies were excluded from the meta-analysis due to lack of appropriate information such as multivariate analysis, adjusted HR, or 95% CI. Further, the high difference in the 95% CI of included studies of the meta-analysis leading to high standard error and different weights in each study, which indicate that the meta-analysis should be interpreted cautiously. Therefore, we suggest more well-designed primary studies to increase the quality of evidence.

## Conclusions

In conclusion, this systematic review and meta-analysis demonstrated that VEGF overexpression in patients with malignant salivary gland neoplasms has prognostic value and is associated with poor overall survival and may be useful in clinical practice.
